# Effect of home‐based hot bathing on exercise‐induced adaptations associated with short‐term resistance exercise training in young men

**DOI:** 10.14814/phy2.70188

**Published:** 2025-01-30

**Authors:** Ryosuke Takeda, Tsubasa Amaike, Taichi Nishikawa, Kohei Watanabe

**Affiliations:** ^1^ Laboratory of Neuromuscular Biomechanics, School of Health and Sport Science Chukyo University Toyota Japan; ^2^ Graduate School of Health and Sport Sciences Chukyo University Toyota Japan

**Keywords:** bathing, cardiovascular system, electrical stimulation, peripheral muscle condition, resistance exercise training

## Abstract

This study investigated whether home‐based bathing intervention (HBBI) improve muscle strength gain and protect cardiovascular function by short‐term resistance training (RT). Thirty‐one healthy young men measured the maximum voluntary isometric contraction (MVC) of knee extensor, electrically evoked knee extension torque, and mean arterial pressure (MAP). Then, participants were divided into three groups with matching MVC: shower without bathing (control, *n* = 10), thermoneutral bathing (36°C‐bathing, *n* = 10), and hot bathing (40°C‐bathing, *n* = 11), and conducted 2 weeks of HBBI. Following familiarization for HBBI, participants completed 2 weeks of HBBI and acute RT (five sessions of three sets of 10 isometric knee extension at 60% MVC). Baseline neuromuscular and cardiovascular function was assessed again following completion of the 2 weeks of intervention. MVC was non‐significantly increased after the RT period in 40°C‐bathing with large effect size (partial *η* ^2^ = 0.450). The electrically evoked knee extension torque (10/100‐Hz ratio) was significantly increased after the RT period in control (*p* = 0.020). MAP did not alter due to bathing intervention and RT (all *p* > 0.05). HBBI improved muscle strength without RT‐induced alteration of peripheral muscle condition. Shower without bathing reduced muscle strength gain but increased peripheral muscle condition. Short‐term RT does not adversely affect the cardiovascular function, regardless of HBBI.

## 1 INTRODUCTION

Resistance exercise training has been widely recommended for individuals of all ages and fitness levels (American College of Sports, 2009). It offers numerous benefits, including increased muscle strength and mass, improved bone density, enhanced metabolic rate, improved joint function, and reduced risks of chronic diseases such as type 2 diabetes and cardiovascular disease (American College of Sports, 2009). A recent systematic review and meta‐analysis of the effect of resistance exercise training suggested that the training volume and training intensity are key factors for improving muscle strength and mass, and power output (Vieira et al., 2021). However, resistance exercise training can affect peripheral muscle conditions, such as inducing peripheral muscle fatigue (Bestwick‐Stevenson et al., 2022), which consequently reduces the training volume (Gorostiaga et al., 2012, 2014), and ultimately, the benefits of training (Vieira et al., 2021). Indeed, our previous study (Nishikawa et al., 2024) demonstrated that performing three sets of 10 isometric knee extensions at 60% of maximum voluntary isometric contraction (MVC) acutely reduced MVC of the knee extensors by approximately 15% and induced peripheral muscle fatigue, as evidenced by an ~22% reduction in electrically elicited twitch torque following a single session of resistance exercise in young adults. Furthermore, fatigue induced by resistance exercise training delays recovery from muscle damage (Vieira et al., 2022) and increases the risk of injury (Ekstrand et al., 2011).

In addition, numerous studies have reported that resistance exercise training at low intensity can improve cardiovascular function such as reduction of blood pressure (BP) (Edwards et al., 2023) and decrease in arterial stiffness (Figueroa et al., 2019). Howden et al. (2002) found that 3 weeks of isometric exercise training of the legs at 20% MVC reduced systolic BP (SBP). On the other hand, Kawano et al. (2008) demonstrated that resistance‐trained men performing moderate‐ to high‐intensity exercises exhibited elevated SBP and increased arterial stiffness, both of which are risk factors for cardiovascular disease (Kawano et al., 2006, 2008; Miyachi et al., 2004). This suggests that moderate‐ to high‐intensity resistance exercise training may modulate cardiovascular function. However, evidence remains limited, particularly regarding short‐term resistance training and potential countermeasures. Therefore, it is crucial to investigate the effect of resistance exercise training on both muscle and cardiovascular function, while also exploring strategies to maximize its benefits for these functions.

Both cold and hot water immersion have long been considered an effective strategy to recover from fatigue after resistance exercise (Versey et al., 2013). Wilcock et al. (2006) reported that thermoneutral and hot water immersion can cause physiological changes within the body, including intracellular‐intravascular fluid shifts, reduction of muscle edema, and increased cardiac output (without increasing energy expenditure), which increases blood flow and possibly nutrient and waste transport throughout the body. Furthermore, several studies have examined the acute effects of hot bathing interventions immediately before or after excessively high‐intensity exercise. Acute hot bathing intervention for ~25–45 min has been shown to effectively aid recovery of muscle strength and reduce muscle damage (Sabapathy et al., 2021; Sautillet et al., 2024). However, for many individuals, replicating the conditions for bathing immediately before or after exercise training, as demonstrated in previous studies (Sabapathy et al., 2021; Sautillet et al., 2024), can be challenging. If home‐based hot bathing interventions, performed without specific time constraints, can produce effects comparable to those observed in these studies, more individuals may adopt them as a strategy to mitigate resistance exercise training‐induced peripheral muscle fatigue and thereby maximize the benefits of resistance training.

The benefits of home‐based hot bathing immersion for resistance exercise training may extend beyond recovery from fatigue. A systematic review and meta‐analysis of the effects of heat therapy on the cardiovascular function reported that heat therapy, such as hot bathing, contributes to the prevention of hypertension and the improvement of arterial stiffness (Pizzey et al., 2021). Thus, a home‐based hot bathing intervention may have a beneficial effect on cardiovascular function, which may be altered by resistance exercise training.

Therefore, the purpose of this study was to examine the effects of a home‐based bathing intervention on short‐term exercise adaptations, including improvements in muscle strength and recovery from peripheral muscle fatigue. In addition, the study aimed to examine the interaction between the home‐based bathing intervention and resistance exercise training on cardiovascular function. We hypothesized that the home‐based hot water bathing intervention would improve muscle strength while promoting recovery from peripheral muscle fatigue and offset the negative effects on the cardiovascular function.

To test our hypothesis, three intervention groups were created: (1) showering without bathing; (2) 36°C bathing, a bathing temperature that does not affect the core temperature, cardiac output, or vascular resistance (Sautillet et al., 2024); (3) 40°C bathing, which was set according to the average daily bathing habits reported in previous studies (Kohara et al., 2018). Vaile et al. (2008) showed that 38°C bathing for 14 min was effective in recovering isometric strength, while Viitasalo et al. showed that 20 min of warm (37°C) underwater jet massage had no significant effect on maximal isometric strength of leg extension (Viitasalo et al., 1995), so we considered that 40°C bathing may become a better strategy to overcome these negative side of resistance exercise training.

## 2 METHODS

### 2.1 Participants

Thirty‐one healthy young men (age: 20.8 ± 0.5 yrs., height: 171.8 ± 6.1 cm, body mass: 62.6 ± 6.8 kg) participated in this study. Sixteen participants were habitual bathers, defined as individuals who bathed at a temperature between 38°C and 42°C, with a frequency of at least five times per week, and for a duration of more than 10 min per session. All participants were healthy with no history of metabolic, musculoskeletal or neurological disorders. They were excluded if they had resistance training habit, current smokers, or their body mass index was >30 kg/m^2^. All participants provided written informed consent, the research ethics committee of Chukyo University approved the study protocol (approval number: 2023‐036), and it was conducted in accordance with the Declaration of Helsinki.

**TABLE 1:** Participant characteristics and effect of bathing and exercise intervention on body mass, appendicular skeletal muscle mass, and comfort level of bathing intervention and subjective recovery from perceived fatigue by training.

	Control, *n* = 10	36°C bathing, *n* = 10	40°C bathing, *n* = 11	*p*‐value
Age, years	20.9 ± 0.6	20.9 ± 0.3	20.7 ± 0.5	Kruskal–Wallis test: *p* = 0.509
Height, cm	175.2 ± 6.7	170.3 ± 5.8	170.1 ± 4.8	One‐way ANOVA: *p* = 0.490
Body mass, kg				Two‐way mixed ANOVA
PRE intervention	66.0 ± 5.0	61.8 ± 7.1	60.3 ± 7.4	*p* = 0.433 (time)
PRE training	65.9 ± 4.8	61.8 ± 6.9	60.4 ± 7.5	*p* = 0.213 (group)
POST training	65.5 ± 5.0	61.6 ± 7.3	60.7 ± 8.0	*p* = 0.706 (time × group)
Appendicular skeletal muscle mass, kg				Two‐way mixed ANOVA
PRE intervention	32.1 ± 2.2	30.8 ± 4.0	29.8 ± 3.1	*p* = 0.790 (time)
PRE training	32.0 ± 1.9	30.7 ± 4.1	29.7 ± 3.2	*p* = 0.368 (group)
POST training	32.1 ± 2.4	30.6 ± 4.3	30.0 ± 3.4	*p* = 0.968 (time × group)
Comfort level of bathing intervention, a.u.	0.25 ± 1.58	−1.17 ± 2.14	1.55 ± 1.69*	Kruskal–wallis test: *p* = 0.006
Subjective recovery from fatigue by training, a.u.	0.75 ± 1.04	0.67 ± 0.82	1.36 ± 0.50*	Kruskal–wallis test: *p* = 0.029

*Note*: Mean ± standard deviation. One‐way analysis of variance (One‐way ANOVA) was used to evaluate the age, height, and questionnaire responses about the comfort level of bathing intervention and subjective recovery from fatigue by training to compare the groups if the normality tests passed. If such tests failed, the Kruskal–Wallis test was used. Two‐way mixed analysis of variance (two‐way mixed ANOVA) was used to evaluate weight and appendicular skeletal muscle mass to compare the groups and time if the normality tests passed.

Abbreviations: a.u., arbitrary units; *n*, number of participants.

* Significantly different versus 36°C bathing group. *p* < 0.05.

### 2.2 Study design and experimental protocol

**FIGURE 1:**
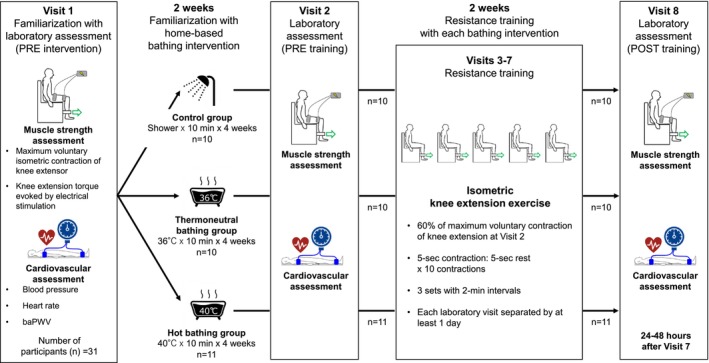
Study design and experimental protocol. BaPWV, brachial‐ankle pulse wave velocity.

Figure 1 shows the study design and protocol. Each participant visited our laboratory eight times. Visit 1 was for screening and familiarization with laboratory assessment before intervention (PRE intervention): assessment of the body composition using a bioimpedance method, as well as voluntary and involuntary knee extension strength and cardiovascular measurements. Following Visit 1, participants underwent a home‐based bathing intervention for the reminder of the study period (after Visit 1 to Visit 8). Participants were divided into three groups according to MVC of the knee extensors: (1) showering without bathing (control group, *n* = 10), thermoneutral bathing (36°C bathing group, *n* = 10), and hot bathing (40°C bathing group, *n* = 11) every day during the intervention. The characteristics of the participants in each group are shown in Table 1. Visit 2 was conducted 2 weeks after Visit 1 for laboratory assessment with the same measurements as in Visit 1 to evaluate the effect of the home‐based bathing intervention on voluntary or involuntary muscle strength and the cardiovascular function. Data from Visit 2 were also used as baseline measurements before resistance exercise training (PRE training). Visits 3–7 were for resistance exercise training, which is described below. Visit 8 was for laboratory assessment with the same measurements as in Visits 1 and 2 to evaluate the interaction effect of resistance exercise training and home‐based bathing intervention on peripheral muscle fatigue and cardiovascular function (POST training). The POST measurement (Visit 8) was conducted 24–48 h after the last bout of resistance exercise to minimize the effects of the last bout of resistance exercise and to evaluate the effects of short‐term resistance exercise training on muscle strength and peripheral muscle condition. After a single bout of resistance exercise, muscle strength and peripheral muscle condition typically recover in ~24 h (Kataoka et al., 2022; Yang et al., 2018). Following the laboratory assessments at Visit 8, participants completed a questionnaire to assess the comfort level of the bathing intervention and their subjective recovery from perceived fatigue by resistance exercise training. The investigators remained blind to the bathing intervention until the questionnaire was administered. On laboratory assessment days (Visits 1, 2, and 8), participants visited our laboratory at the same time of day (10:00 a.m.). Participants were asked to maintain their regular daily routine and to eat the same or similar meals 1 h before their visit to the laboratory. They were asked to refrain from alcohol and caffeine 12 h before their visit to the laboratory for assessment and avoid vigorous exercise 24 h before the day of laboratory assessment.

### 2.3 Bathing intervention

After Visit 1, the control group only showered for 10 min. There were two habitual bathers in the control group. To control the bathing temperature, participants with bathtubs equipped with automatic temperature controls were recruited, and the water temperature was maintained at 36°C and 40°C throughout the bathing sessions. For the 36°C bathing group, bathing was performed at 36°C for 10 min. There were six habitual bathers in the 36°C bathing group. In the 40°C bathing group, bathing was performed at 40°C for 10 min. There were eight habitual bathers in the 40°C bathing group. The bathing intervention took place daily for 4 weeks (from after Visit 1 to Visit 8). Participants in both bathing groups sat in the Japanese style bathtub and immersed themselves in the water up to heart level. Bathing temperature during immersion and timing of bathing were not controlled.

### 2.4 Resistance exercise training

Participants performed isometric knee extension resistance exercise training five times over a 2‐week period in our laboratory. This protocol was chosen to examine acute adaptations without the confounding influence of skeletal muscle hypertrophy that typically accompanies chronic training. Each laboratory visit was separated by at least 1 day. Exercise mode was determined based on our previous study (Nishikawa et al., 2024). Participants were seated on a custom‐made chair to perform resistance exercise training with hip and knee angles at 90 degrees. A dynamometer (Takei Scientific Instruments Co., Ltd., Niigata, Japan) and a force transducer (LU‐100KSE; Kyowa Electronic Instruments, Aichi, Japan) were attached to the leg of the chair. Participants performed three sets of 10 isometric knee extensions with the right leg, with a rest period of 120 seconds between sets. Intensity was set at 60% MVC of the knee extensor, which was measured during Visit 2. A contraction consisted of a 5‐sec rest phase and a 5‐sec sustained phase at the target torque. A metronome was used to be able to clearly identify the contraction time. Visual feedback of the target force was provided on a personal computer monitor during these submaximal contractions to allow participants to exert force at the target level. All participants fully completed the resistance exercise tasks.

### 2.5 Measurements

#### 2.5.1 Maximum knee extension torque

Participants were seated in a custom‐made chair to perform maximum voluntary isometric knee extension with hip and knee angles at 90 degrees. A dynamometer (Takei Scientific Instruments Co., Ltd., Niigata, Japan) and a force transducer (LU‐100KSE; Kyowa Electronic Instruments, Aichi, Japan) were attached to the leg of the chair. Participants performed two to three MVCs of the knee extensors with at least 30 sec intervals between each trial, following warm‐up sessions consisting of efforts ranging from 30% to 90% of maximal effort. Participants were instructed to maintain the MVC force for 3 sec, and the steady state force produced was averaged to calculate the MVC torque. MVC torque was calculated by multiplying the force value by the distance between the knee joint axis and the force transducer placed on the distal part of the right shank. The strongest MVC for each measurement was selected for further analysis as the MVC torque at that given time.

#### 2.5.2 Evoked knee extension torque by electrical stimulation

Electrical stimulation was used to induce knee extensor contraction, which was measured to estimate involuntary muscle strength using a constant current stimulator (DS7AH, Digitimer Ltd., Hertfordshire, UK). Two carbon rubber electrodes (4.5 × 28 cm) were attached in proximal and distal regions of the quadriceps femoris according to a previous study (Hirono et al., 2024). The electrodes covered the proximal regions of the rectus femoris and vastus lateralis and the distal regions of the rectus femoris, vastus lateralis, and medialis. Electrical stimulation was applied through these electrodes with a 50‐μsec pulse width, and the intensity was increased by 10 mA until the evoked torque reached a plateau to evaluate the maximum twitch torque. The maximum current intensity was increased by a further 20% (i.e., supramaximal) and held constant for all subsequent tests. Singlet and doublet stimuli at 10 and 100 Hz were measured, and the 10/100‐Hz ratio was calculated to assess peripheral muscle condition, and the half‐relaxation time (time to 1/2 peak torque from time to peak torque) was calculated to assess peripheral muscle fatigue (Lievens et al., 2020). At subsequent visits, the electrodes could be reapplied in the same region using an ink mark on the thigh. The maximum current intensity was re‐determined at each measurement on Visits 1, 2, and 8.

#### 2.5.3 Cardiovascular measurements

Heart rate (HR), systolic and diastolic BP (SBP and DBP, respectively), and brachial‐ankle pulse wave velocity (baPWV), an index of systemic arterial stiffness (Sugawara et al., 2005), were measured at least 5 min after adopting a supine resting position using the validated and fully automated PWV/ABI analyzer (HBP‐8000, OMRON, Kyoto, Japan). Bilateral brachial and post‐tibial arterial pressure waveforms were stored for ~1 min by extremity cuffs wrapped around both upper arms and ankles that were connected to an oscillometric pressure sensor. BaPWV was calculated for the left and right sides using the distance between the two upper and lower arterial recording sites divided by their respective transit times. Again, the transit time was determined from the time delay between the proximal and distal foot waveforms. Higher values of baPWV were used for assessment. Mean arterial pressure (MAP) was calculated as (SBP‐DBP)/3 + DBP.

#### 2.5.4 Body composition

An eight‐polar impedance meter (InBody 430, InBody Japan Inc., Tokyo, Japan) was used to evaluate the appendicular muscle mass of each body segment. Resistance (*R*) of arms and legs was measured at frequencies of 5, 50, 250, and 500 kHz with eight tactile electrodes: two in contact with the palm and thumb of each hand and two in contact with the anterior and posterior aspects of the sole of each foot (Malavolti et al., 2003). The subject stands with the soles of the feet in contact with the foot electrodes and grasps the hand electrodes. The sequence of measurements, controlled by a microprocessor, proceeds as follows. An alternating current (a.c.) of 250 μA was applied, and the recorded voltage difference (V) was used to calculate the resistance for each body segment. Segmental resistance indices (RI) were calculated as Height (cm)^2^/*R*_x_ (Ω), where *R*_x_ is the resistance of the arm or leg at a given frequency. Resistance in appendicular muscle (*R*_sumx_) was calculated as the sum of segmental Rx (right arm + left arm + right leg + left leg). Resistance index (RI_sumx_) in the appendicular muscle was calculated as height (cm)^2^/R_sumx_ (Ω). Water intake was ad libitum, and measurements were taken after urination.

#### 2.5.5 Questionnaire about the comfort level of bathing intervention

After the POST measurement (Visit 8), participants were administered a questionnaire about the comfort level of bathing intervention, with scoring as follows: ‐3, very uncomfortable; ‐2, uncomfortable; ‐1, slightly uncomfortable; 0, neither comfortable nor uncomfortable; 1, slightly comfortable; 2, comfortable; 3, very comfortable.

#### 2.5.6 Questionnaire about subjective recovery from perceived fatigue resistance exercise training

After the POST measurement (Visit 8), participants were given a questionnaire about subjective recovery from resistance exercise training‐induced fatigue caused by bathing, with scoring as follows: 0, no recovery from fatigue; 1, low‐level recovery from fatigue; 2, recovery from fatigue; 3, marked recovery from fatigue.

### 2.6 Statistical analyses

Sample size was determined using G*Power Version 3.1.9.6. It was determined based on our preliminary data on the 10/100‐Hz ratio before and after resistance exercise training with shower‐only intervention (mean value with standard deviation (SD) of PRE training was 0.714 with 0.111, and the mean value with SD of POST training was 0.976 with 0.157) to ensure sufficient power at the neuromuscular fatigue level. The value *n* = 8 will provide 95% power at an α‐level of 0.05. Considering the possibility that participants may drop out, we enrolled at least 10 participants in each group to ensure sufficient power.

Statistical analyses were performed using IBM SPSS v. 25. Values are expressed as the mean ± SD. One‐way analysis of variance (one‐way ANOVA) was used to evaluate the age, height, and questionnaire responses if these data in each group passed the normality tests using Shapiro–Wilk tests. As age in all groups (all *p* < 0.050), comfort level of bathing intervention in all groups (all *p* < 0.050), and subjective recovery from fatigue by training in both bathing groups (*p* < 0.050, respectively) failed the normality tests, the Kruskal–Wallis test was used to compare variables among groups. Other variables between group (control vs. 36°C bathing vs. 40°C bathing) and time (PRE intervention vs. PRE training vs. POST training) were compared using two‐way mixed analysis of variance (two‐way mixed ANOVA) if these data in each group at each time passed the normality tests using Shapiro–Wilk tests. Effect sizes [partial eta squared (partial *η* ^2^)] were calculated when appropriate. As the half‐relaxation time at a singlet stimulus in 40°C bathing group at POST training and the half‐relaxation time at a doublet stimulus at 100 Hz in 36°C bathing group at PRE intervention failed the normality tests (*p* = 0.037 and *p* = 0.028, respectively), the Kruskal–Wallis test was applied to compare variables between the groups at each time and the Friedman test to compare variables between times within each group. Bonferroni‐corrected post‐hoc procedures were used when appropriate. Pearson's correlation (r) was calculated to examine the relationship between changes in MVC following resistance exercise training and alterations in half relaxation time at doublet stimulus at 10 Hz and 100 Hz, as these data passed the normality tests using the Shapiro–Wilk tests. Conversely, Spearman's correlation (r_s_) was used to evaluate the relationship between changes in MVC with resistance exercise training and alterations in half relaxation time at singlet stimulus as well as between subjective recovery from perceived fatigue with resistance exercise training and other variables, as the half relaxation time at singlet stimulus and subjective recovery from perceived fatigue with resistance exercise training failed the normality tests (*p* = 0.040 and *p* = 0.001, respectively). A *p*‐value of <0.05 was considered significant.

## 3 RESULTS

Resistance exercise training and bathing intervention did not alter the body mass or appendicular skeletal muscle mass (time and interaction, all *p* > 0.05). The comfort level of bathing intervention was significantly higher in the 40°C than 36°C bathing group (post‐hoc test, *p* = 0.004), but there was no significant difference among the control and both bathing groups (*p* > 0.05). Subjective recovery from perceived fatigue by resistance exercise training was significantly higher in the 40°C than 36°C bathing group (post‐hoc test, *p* = 0.049), but there was no significant difference among control and both bathing groups (*p* > 0.05).

### 3.1 Voluntary and involuntary muscle strength assessment

**FIGURE 2:**
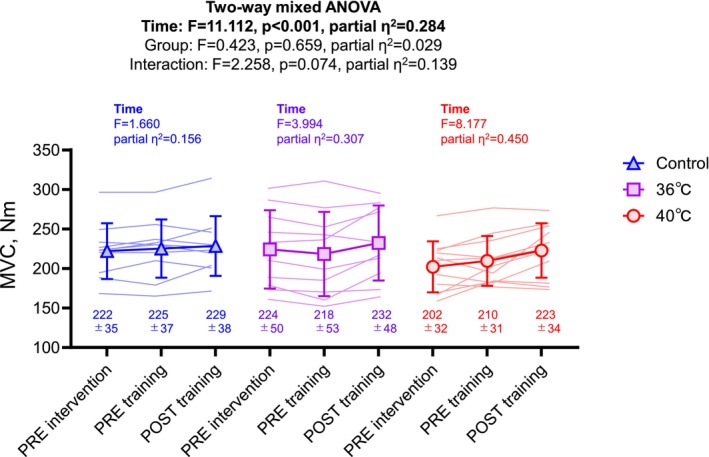
Maximum voluntary isometric contraction (MVC) of the knee extensor PRE bathing intervention, PRE and POST resistance exercise training with home‐based bathing intervention in shower‐only (control), thermoneutral bathing (36°C), and hot bathing (40°C) groups. The triangles, squares, and circles denote the control, 36°C, and 40°C groups, respectively. Two‐way mixed analysis of variance (Two‐way mixed ANOVA) was used to compare the groups and time if the normality tests passed. Values are mean ± standard deviation.

Figure 2 shows the effect of interaction between resistance exercise training and intervention on MVC of the knee extensor. Although there was no significant interaction (*p* = 0.074, partial *η* ^2^ = 0.139), MVC of the knee extensor was increased after resistance exercise training with a large effect size in the 40°C group (partial *η* ^2^ = 0.450), a medium effect size in the 36°C group (partial *η* ^2^ = 0.307), and a small effect size in the control group (partial *η* ^2^ = 0.156). There was no significant difference among groups.

Table 2 shows the effect of interaction between resistance exercise training and intervention on the knee extension torque evoked by electrical stimulation. Responses to the singlet stimulus were not significantly different across groups, time points, or their interaction (all *p* > 0.05), and the effect size of time was small in all groups (partial *η* ^2^ = 0.194 in control group, 0.091 in 36°C group, 0.145 in 40°C group). Responses to the doublet stimulus at 10 Hz showed a trend toward an increase following resistance exercise training (*p* = 0.091) with a medium effect size in the control group (partial *η* ^2^ = 0.349), but the effect sizes were small in the 36°C group (partial *η* ^2^ = 0.065) and the 40°C group (partial *η* ^2^ = 0.168). However, there were no significant differences among groups (*p* = 0.893, partial *η* ^2^ = 0.008) or in the group × time interaction (*p* = 0.240, partial *η* ^2^ = 0.092). Responses to the doublet stimulus at 100 Hz were not significantly different across groups, time points, or their interaction (all *p* > 0.05), with the effect size of time remaining small in all groups (partial *η* ^2^ = 0.040 in the control group, 0.044 in the 36°C group, and 0.169 in the 40°C group). Half relaxation time at singlet stimulus was increased after resistance exercise training only in the 36°C group (*p* = 0.048) but no significant group difference and interaction. Half relaxation time at 10 or 100 Hz doublet stimulus were not significantly different among group, time, and their interaction (all *p* > 0.05).

**TABLE 2:** The effect of interaction between resistance exercise training and intervention on knee extension torque evoked by electrical stimulation.

	Control, *n* = 10	36°C bathing, *n* = 10	40°C bathing, *n* = 11	*F*‐value, *p*‐value, effect size	
Singlet stimulus at 1 Hz, Nm				Two‐way mixed ANOVA	
PRE intervention	24.0 ± 2.9	24.3 ± 8.5	23.4 ± 6.1	Time: *F* = 2.084, *p* = 0.134, partial *η* ^2^ = 0.069	
PRE training	25.6 ± 8.6	25.8 ± 9.0	26.2 ± 7.4	Group: *F* = 0.163, *p* = 0.850, partial *η* ^2^ = 0.012	
POST training	28.4 ± 6.1	26.4 ± 9.3	23.7 ± 7.6	Time × Group: *F* = 0.903, *p* = 0.469, partial *η* ^2^ = 0.061	
Singlet stimulus Half‐relaxation time, msec				Friedman test	Kruskal–wallis test
PRE intervention	81.9 ± 15.2	68.1 ± 22.7	72.4 ± 15.2	*p* = 0.584 (control)	*p* = 0.210 (PRE intervention)
PRE training	78.7 ± 18.3	71.0 ± 18.4	75.0 ± 12.6	*p* = 0.048 (36°C bathing)	*p* = 0.387 (PRE training)
POST training	82.5 ± 13.1	78.7 ± 16.6	79.0 ± 11.6	*p* = 0.534 (40°C bathing)	*p* = 0.719 (POST training)
Doublet stimulus at 10 Hz, Nm				Two‐way mixed ANOVA	
PRE intervention	37.2 ± 5.7	36.8 ± 16.6	36.0 ± 8.6	Time: *F* = 2.502, *p* = 0.091, partial *η* ^2^ = 0.082	
PRE training	38.7 ± 9.2	39.0 ± 15.3	40.7 ± 10.9	Group: *F* = 0.113, *p* = 0.893, partial *η* ^2^ = 0.008	
POST training	43.5 ± 8.8	41.0 ± 16.0	36.2 ± 11.2	Time × Group: *F* = 1.417, *p* = 0.240, partial *η* ^2^ = 0.092	
Doublet stimulus at 10 Hz Half‐relaxation time, msec				Two‐way mixed ANOVA	
PRE intervention	69.7 ± 12.2	70.8 ± 17.1	68.5 ± 11.8	Time: *F* = 1.348, *p* = 0.268, partial *η* ^2^ = 0.046	
PRE training	73.7 ± 12.9	70.6 ± 15.6	70.1 ± 13.4	Group: *F* = 0.096, *p* = 0.909, partial *η* ^2^ = 0.007	
POST training	72.3 ± 12.8	73.3 ± 10.5	70.6 ± 11.2	Time × Group: *F* = 0.418, *p* = 0.795, partial *η* ^2^ = 0.029	
Doublet stimulus at 100 Hz, Nm				Two‐way mixed ANOVA	
PRE intervention	46.0 ± 6.4	46.9 ± 14.1	45.0 ± 8.9	Time: *F* = 1.287, *p* = 0.284, partial *η* ^2^ = 0.044	
PRE training	46.3 ± 7.8	49.5 ± 14.8	49.5 ± 12.1	Group: *F* = 0.140, *p* = 0.870, partial *η* ^2^ = 0.010	
POST training	47.7 ± 8.1	49.6 ± 14.2	45.3 ± 10.4	Time × Group: *F* = 0.680, *p* = 0.608, partial *η* ^2^ = 0.046	
Doublet stimulus at 100 Hz Half‐relaxation time, msec				Friedman test	Kruskal–wallis test
PRE intervention	72.4 ± 11.3	74.1 ± 19.4	71.9 ± 21.1	*p* = 0.301 (control)	*p* = 0.796 (PRE intervention)
PRE training	73.1 ± 15.6	65.9 ± 13.0	68.8 ± 14.6	*p* = 0.836 (36°C bathing)	*p* = 0.420 (PRE training)
POST training	71.9 ± 21.1	68.8 ± 14.6	73.2 ± 16.9	*p* = 0.977 (40°C bathing)	*p* = 0.245 (POST training)

*Note*: Mean ± standard deviation. Two‐way mixed analysis of variance (two‐way mixed ANOVA) was used to evaluate each measurement to compare the groups and time if the normality tests passed. If such tests failed, the Friedman test was used to compare the groups and Kruskal–Wallis test was used to compare the time. *p* < 0.05.

Abbreviation: *n*, number of participants.

**FIGURE 3:**
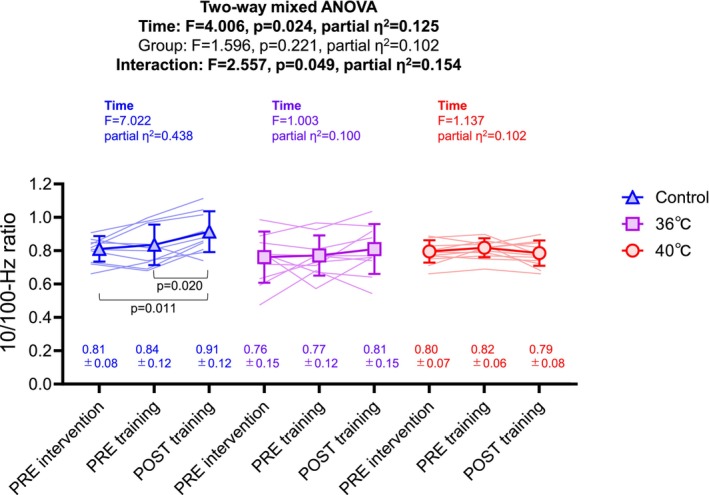
The 10/100‐Hz ratio on PRE bathing intervention and PRE and POST resistance exercise training with home‐based intervention in shower‐only (control), thermoneutral bathing (36°C), and hot bathing (40°C) groups. The triangles, squares, and circles denote the control, 36°C, and 40°C groups, respectively. Two‐way mixed analysis of variance (two‐way mixed ANOVA) was used to compare the groups and time if the normality tests passed. Values are mean ± standard deviation.

The 10/100‐Hz ratio was not significantly different after bathing intervention in any group (Figure 3. all *p* > 0.05). After the resistance exercise training, there was no significant difference in the 36°C group (*p* > 0.05, partial *η* ^2^ = 0.100) or 40°C group (*p* > 0.05, partial *η* ^2^ = 0.102), but the 10/100‐Hz ratio in the control group was significantly increased from PRE resistance exercise training (*p* = 0.020, partial *η* ^2^ = 0.438). The 10/100‐Hz ratio at POST resistance exercise training showed a tendency to be higher in the control compared with 40°C group (*p* = 0.059).

### 3.2 Relationship between muscle strength gain and alteration of peripheral muscle condition after resistance exercise training, and recovery from perceived fatigue by bathing

Change in muscle strength from PRE training to POST training was not associated with alteration of half relaxation time at any stimulus (*p* = 0.412, *r*_s_ = −0.153 in singlet stimulus; *p* = 0.213, *r* = −0.230 in doublet stimulus at 10 Hz; *p* = 0.212, *r* = −0.231 in doublet stimulus at 100 Hz) and alteration of 10/100‐Hz ratio (*p* = 0.123, *r* = −0.283) but positively associated with subjective recovery from perceived fatigue by resistance exercise training (*p* = 0.016, *r*_s_ = 0.444). Subjective recovery from perceived fatigue by resistance exercise training was not associated with alteration of half relaxation time at any stimulus (*p* = 0.624, *r*_s_ = −0.095 in singlet stimulus; *p* = 0.080, *r*_s_ = −0.331 in doublet stimulus at 10 Hz; *p* = 0.280, *r*_s_ = −0.207 in doublet stimulus at 100 Hz) and alteration of 10/100‐Hz ratio (*p* = 0.076, *r*_s_ = −0.335).

### 3.3 Cardiovascular assessment

Table 3 shows the effect of interaction between resistance exercise training and intervention on the cardiovascular function. MAP, HR, and baPWV did not significantly alter with intervention, resistance exercise training, nor their interactions (all *p* > 0.05).

**TABLE 3:** The effect of interaction between resistance exercise training and intervention on each cardiovascular assessment.

	Control, *n* = 10	36°C bathing, *n* = 10	40°C bathing, *n* = 11	*F*‐value, *p*‐value, effect size
MAP, mmHg				Two‐way mixed ANOVA
PRE intervention	85 ± 4	85± 5	83 ± 6	Time: *F* = 3.017, *p* = 0.057, partial *η* ^2^ = 0.097
PRE training	85 ± 5	84 ± 5	82 ± 5	Group: *F* = 1.330, *p* = 0.281, partial *η* ^2^ = 0.087
POST training	82 ± 4	85 ± 5	80 ± 3	Time × Group: *F* = 1.119, *p* = 0.357, partial *η* ^2^ = 0.074
Heart rate, beats/min				Two‐way mixed ANOVA
PRE intervention	64 ± 7	65 ± 8	68 ± 9	Time: *F* = 2.854, *p* = 0.066, partial *η* ^2^ = 0.093
PRE training	63 ± 8	62 ± 6	64 ± 4	Group: *F* = 0.113, *p* = 0.552, partial *η* ^2^ = 0.082
POST training	64 ± 4	65 ± 8	67 ± 5	Time × Group: *F* = 0.335, *p* = 0.853, partial *η* ^2^ = 0.023
BaPWV, cm/sec				Two‐way mixed ANOVA
PRE intervention	1151 ± 119	1149 ± 126	1124 ± 78	Time: *F* = 0.951, *p* = 0.393, partial *η* ^2^ = 0.035
PRE training	1158 ± 122	1163 ± 120	1126 ± 71	Group: *F* = 0.131, *p* = 0.878, partial *η* ^2^ = 0.010
POST training	1184 ± 145	1167 ± 111	1093 ± 60	Time × Group: *F* = 1.774, *p* = 0.148, partial *η* ^2^ = 0.120

*Note*: Mean ± standard deviation. Two‐way mixed analysis of variance (two‐way mixed ANOVA) was used to evaluate each cardiovascular assessment to compare the groups and time. *p* < 0.05.

Abbreviations: BaPWV, brachial‐ankle pulse wave velocity; MAP, mean arterial pressure; *n*, number of participants.

## 4 DISCUSSION

The main findings of this study were as follows: (1) after short‐term resistance exercise training, voluntary muscle strength showed an increase, with a small effect size in the home‐based shower intervention group (partial *η* ^2^ = 0.156), a medium effect size in the home‐based bathing intervention group at thermoneutral temperature (36°C, partial *η* ^2^ = 0.307), and a large effect size in the home‐based bathing intervention group at hot temperature (40°C, partial *η* ^2^ = 0.450) although there were no significant differences between groups; (2) 10/100‐Hz ratio was increased after short‐term resistance exercise training with home‐based shower intervention but not with home‐based bathing intervention at either temperature; (3) subjective comfort and recovery from perceived fatigue were reported to be better with hot compared with thermoneutral bathing; (4) cardiovascular function was not affected by short‐term resistance exercise training with any of the bathing interventions. These results suggest that either hot or thermoneutral home‐based bathing intervention with short‐term resistance exercise training can increase the voluntary muscle strength without inducing changes in peripheral muscle condition. In contrast, shower without bathing associated with reduced muscle strength gains but an improvement in peripheral muscle condition. Furthermore, hot bathing provides subjectively better comfort and recovery from perceived fatigue compared to thermoneutral bathing in healthy young men. However, the cardiovascular function remains unaffected by resistance exercise training combined with any form of bathing intervention in healthy young men.

### 4.1 Effect of home‐based bathing intervention with short‐term resistance exercise training on increase in voluntary muscle strength

In the present study, there were mild to large increases in voluntary muscle strength following short‐term resistance exercise training with a home‐based bathing intervention at either thermoneutral (36°C) or hot (40°C) temperatures. Watanabe et al. (2021) previously reported that short‐term isometric exercise training (three sets of 10 contractions at 80%MVC) can improve muscle strength in young and older individuals (Watanabe et al., 2020). Furthermore, it has been well‐established that short‐term resistance exercise training can induce neural adaptations (Sale, 1988), resulting in an increases in muscle strength even in the absence of increases in muscle size (Akima et al., 1999; Moritani & deVries, 1979; Pearcey et al., 2021). Although Balshaw et al. (2017) reported that pre‐training muscle strength also contributed to muscle strength gains after resistance exercise training, PRE training muscle strength did not differ between groups (Figure 2). Thus, neural adaptation may have contributed to the short‐term resistance exercise training‐induced muscle strength gains in the 36°C and 40°C bathing groups. Importantly, this phenomenon was minimal in the control group. Although we cannot determine whether the muscle strength gains after short‐term resistance exercise training with the bathing intervention were due to neural adaptation, another contributing factor may be the increased muscle blood flow due to bathing. Rodrigues et al. (2022) suggested that an increase in muscle blood flow, and consequently in muscle fluid, may improve muscle contractile function in two ways: by promoting cross‐bridge formation and by increasing muscle stiffness. Muscle strength gains have also been observed after heat acclimation through passive heating (Racinais et al., 2017), suggesting that hot water immersion may not only acutely but also chronically enhance muscle strength during resistance training.

Interestingly, the change in muscle strength from PRE training to POST training was positively associated with subjective recovery from perceived fatigue by resistance exercise training (*p* = 0.016, *r*_s_ = 0.444). As an increase in perceived fatigue is associated with a decrease in MVC (Whittaker et al., 2019), this finding is not surprising. More importantly, this subjective recovery was not associated with any peripheral muscle condition. Previous research has demonstrated that post‐resistance exercise hot water immersion improves perceived recovery and increases circulating testosterone levels (Horgan et al., 2023). Sikorski et al. (2013) reported a positive relationship between free testosterone levels and perceived recovery following high‐volume resistance training. In addition, testosterone administration has been shown to increase muscle mass and strength in a dose‐dependent manner in young men (Bhasin et al., 2001). Thus, the improved subjective recovery from perceived fatigue observed in the 40°C bathing intervention group may be related to an increase in circulating testosterone, which could potentially contribute to improved muscle strength over time. However, it is important to note that the relationship between circulating testosterone levels and peripheral muscle recovery remains unclear. Furthermore, we did not measure testosterone levels in this study, and our interpretation is therefore speculative. Although this explanation is consistent with previous findings, future research should directly measure testosterone levels and assess both central and peripheral fatigue to validate the proposed mechanism and clarify the discrepancy between subjective recovery and objective measures of recovery.

We also initially speculated that peripheral muscle fatigue might impede muscle strength gains due to reduced exertion capacity (Behm et al., 2001; Millet et al., 2003). However, the half relaxation time did not differ between groups. Thus, the increased voluntary muscle strength observed in the bathing group may not be due to recovery from peripheral muscle fatigue.

### 4.2 Effect of peripheral muscle conditions induced by short‐term resistance exercise training with home‐based bathing intervention

It is well known that assessment of the muscle contractile properties using electrical stimulation is a favorable approach to evaluate peripheral muscle conditions (Allman & Rice, 2004; Binder‐Macleod et al., 1998). In the present study, an increase in the 10/100‐Hz ratio was observed only in the control group. Additionally, although no significant group differences were detected, the doublet stimulus responses at 10 Hz in the control group showed a trend toward an increase following resistance exercise training, with a medium effect size (partial *η* ^2^ = 0.349). In contrast, the responses at 100 Hz did not significantly increase, showing only a small effect size (partial *η* ^2^ = 0.040). Interestingly, previous studies have reported that increasing intracellular Ca^2+^ concentration ([Ca^2+^]i) can improve muscle contractile properties. Indeed, similar increases in the 10 Hz doublet have been reported following caffeine consumption (Lopes et al., 1983), which may increase Ca^2+^ sensitivity in sarcoplasmic reticulum (SR) Ca^2+^ release channels and subsequently increase [Ca^2+^]i through Ca^2+^ release from the SR (Stephenson, 2008). Additionally, post‐tetanic potentiation, a transient enhancement of muscle twitch force observed immediately after high‐intensity exercise, has been partially attributed to increased [Ca^2+^]i and increased Ca^2+^ sensitivity induced by muscle contractions (Lochynski et al., 2021). Thus, the observed increase in the 10/100‐Hz ratio and the trend toward increased doublet stimulus responses at 10 Hz in the control group may reflect training‐induced adaptations related to improvements of low‐frequency muscle function, such as increased [Ca^2+^]i levels and enhanced Ca^2+^ sensitivity.

Interestingly, these phenomena were not observed in the bathing groups. While acute hot bathing interventions have been shown to aid in muscle strength recovery and reduce muscle damage (Sabapathy et al., 2021; Sautillet et al., 2024) and may help maintain [Ca^2+^]i homeostasis (Ikegami et al., 2021; Rodrigues et al., 2022), these effects of bathing may reduce the magnitude of the stimulus required for exercise‐induced adaptations related to improvements in low‐frequency muscle function. Indeed, emerging evidence suggests that seemingly beneficial interventions, such as antioxidant consumption, can attenuate training‐induced adaptations by reducing reactive oxygen species (ROS), which act as intracellular signaling molecules to promote such adaptations (Merry & Ristow, 2016). Importantly, Merry and Ristow suggested that lower doses of stressors, such as reactive oxygen and nitrogen species (ROS/RNS), exert beneficial effects, whereas theoretical high exposure to ROS/RNS—though rarely achieved during training—could impair training adaptations and performance (Merry & Ristow, 2016). Future studies are needed to determine whether bathing attenuates the signaling mechanisms for exercise‐induced adaptations and to explore whether bathing could be beneficial for exercise‐induced adaptations at higher training intensities than those used in the present study.

### 4.3 Effect of home‐based bathing intervention on cardiovascular function by short‐term resistance exercise training

In contrast to our hypothesis, BP, HR, and baPWV were not altered by short‐term resistance exercise training even in the control group. This may be due to differences in the duration of exercise training (Kawano et al., 2006), age range of participants (Miyachi et al., 2004), or training intensity (Miyachi et al., 2004), although a systematic review and meta‐analysis suggested that the exercise intensity was not associated with BP when the duration of resistance exercise training intervention was short or medium term (Cornelissen & Smart, 2013). Further studies are needed to investigate whether home‐based bathing intervention can minimize the resistance exercise training‐induced cardiovascular risk.

### 4.4 Difference in benefit of 40°C versus 36°C bathing intervention

In the present study, muscle strength gain was large in the 40°C bathing intervention and mild in the 36°C bathing intervention but there was no significant difference in the change in peripheral muscle condition, or cardiovascular function after short‐term resistance exercise training. Although we did not assess cardiac output or vascular resistance, a previous study reported that an increased cardiac output and decreased vascular resistance, which can increase blood flow and possibly nutrient and waste transport throughout the body (Wilcock et al., 2006), wase observed in the 40°C bathing group but not in the 36°C bathing group. More importantly, subjective comfort and recovery from fatigue were reported to be more favorable with 40°C bathing compared to 36°C bathing (Table 1). Furthermore, as the associated risk of heat illness is not high with 40°C bathing for 10 min because of only a mild increase in the core temperature (Sautillet et al., 2024), we recommend daily bathing at 40°C rather than 36°C to improve muscle strength and suggest a positive effect on the subjective recovery from perceived fatigue.

### 4.5 Experimental considerations

There are several experimental considerations. First, we did not measure voluntary activation. Although central fatigue was a minor contributor to force reduction compared to peripheral muscle fatigue (Kataoka et al., 2022), future studies should assess voluntary activation to better understand whether the increase in muscle strength from short‐term resistance training is due to neural adaptations or other factors. Second, we did not assess the peripheral muscle condition such as 10/100‐Hz ratio or muscle strength immediately after the resistance exercise task. However, a previous study (Dalton et al., 2024) showed that the 10/100‐Hz ratio decreased immediately after the fatiguing isometric exercise and another previous study (Kamandulis et al., 2019) showed that the 20/100‐Hz ratio, which is similar to the 10/100‐Hz used in our study, decreased after the fatiguing drop jump exercise and recovered to the baseline level need after 2–3 days. Therefore, all participants in the present study could be induced ~20% depression of 10/100‐Hz immediately after the resistance training, as reported by Dalton et al. (2024). Third, although we adopted the exercise protocol of three sets of 10 isometric knee extensions at 60% MVC from previous research (D'Souza et al., 2023; Kawano et al., 2008; Nishikawa et al., 2024), this may not have been sufficient to induce significant muscle fatigue, neuromuscular adaptations, or affect the cardiovascular function. However, the key considerations for selecting the exercise mode were as follows: (1) the exercise mode should affect muscle strength and/or peripheral muscle condition, and (2) all participants should be able to complete the exercise protocol with a matched exercise volume. In fact, all participants were able to complete all exercise training sessions. Fourth, we did not control for the timing of the bathing intervention. Although we believe that bathing within 24 h of training is sufficient, as previous studies have shown that recovery of MVC and the 10/100‐Hz ratio to baseline levels takes over 2–3 days without bathing intervention (Kamandulis et al., 2019; Sautillet et al., 2024), we cannot exclude the possibility that the timing of bathing may have influenced recovery as well as the post‐intervention testing results.

## 5 CONCLUSION

The home‐based bathing intervention, particularly bathing at 40°C, promoted muscle strength gains and improved subjective recovery from perceived fatigue but did not enhance peripheral muscle condition following resistance exercise training. Interestingly, alterations in peripheral muscle condition were observed only in the showering group in response to resistance exercise training. On the other hand, short‐term resistance exercise training does not adversely affect the cardiovascular function regardless of bathing habits.

## AUTHOR CONTRIBUTIONS

R.T. contributed to conception and design of the experiments, correction, analysis, and interpretation of data, and drafting of the article and revising it critically for important intellectual content. K.W. contributed to conception and design of the experiments, revising the article for important intellectual content, and obtained funding support. T.A. contributed to correction and analysis of data and revising the article. T.N. contributed to correction of data and revising the article. All authors approved the final version of the manuscript.

## FUNDING INFORMATION

This work was supported by Rinnai Corporation.

## CONFLICT OF INTEREST STATEMENT

The authors declare no conflicts of interest.

## ETHICS STATEMENT

All participants provided written informed consent, the research ethics committee of Chukyo University approved the study protocol (approval number: 2023‐036), and it was conducted in accordance with the Declaration of Helsinki.

## Data Availability

Data that support the findings of the present study are presented in the text, figures, and table, and are available from the corresponding author upon reasonable request.
